# Acute respiratory symptoms and its associated factors among mothers who have under five-years-old children in northwest, Ethiopia

**DOI:** 10.1186/s12199-020-00859-4

**Published:** 2020-06-15

**Authors:** Zewudu Andualem, Zelalem Nigussie Azene, Jember Azanaw, Asefa Adimasu Taddese, Henok Dagne

**Affiliations:** 1 Department of Environmental and Occupational Health and Safety, Institute of Public Health, College of Medicine and Health Sciences, University of Gondar, Gondar, Ethiopia; 2 Department of Women’s and Family Health, School of Midwifery, College of Medicine and Health Sciences, University of Gondar, Gondar, Ethiopia; 3 Department of Epidemiology and Biostatistics, Institute of Public Health, College of Medicine and Health Sciences, University of Gondar, Gondar, Ethiopia

**Keywords:** Children, Mothers, Household air pollution, Respiratory symptoms, Ethiopia

## Abstract

**Background:**

Poor air quality of the household is likely to be the largest public health concern in resource-constrained countries. Exposure to household air pollution, poor working environment, and fuel type used at household level have been associated with respiratory symptoms. This study aimed to assess acute respiratory symptoms and its associated factors among mothers who have under five-years-old children in Gondar city, northwest Ethiopia.

**Method:**

A cross-sectional study was carried out from February 15, 2019 to June 20, 2019. Study participants were selected using simple random sampling, and data were collected via face-to-face interviews. Binary logistic regression analysis was used to test the association of explanatory and outcome variables. Variables with *p* < 0.05 were considered as significantly associated with the outcome variable.

**Results:**

The prevalence of respiratory symptoms among mothers of under-five years-old children in this study was 46.1%. Education (vocational training compared to cannot read and write) (adjusted odd ratio (AOR) = 0.26 at 95% confidence interval (CI): 0.08–0.82), working in dusty environment (AOR = 2.90 at 95% CI: 1.39–6.08), wood fire use (AOR = 0.37 at 95% CI: 0.16–0.85), living in mud- and wood-walled houses (AOR = 0.53 at CI: 0.32–0.89), recent house painting (AOR = 1.95 at 95% CI: 1.03–3.69), new carpet (AOR = 2.02 at 95% CI: 1.08–3.77), pesticide use (AOR = 1.71 at 95% CI: 1.03–2.84), damp stain (AOR = 2.45 at 95% CI: 1.04–5.75), spending longer time in house for 6 to 11 h (AOR = 2.59 at 95% CI: 1.53–4.37) and 11 to 15 h (AOR = 3.47 at 95% CI: 1.87–6.43), and living less than 100 m from unpaved roads/streets (AOR = 4.35 at 95% CI: 2.64–7.18) were significantly associated with respiratory symptoms among mothers of under-five years-old children.

**Conclusion:**

Respiratory symptoms were common among mothers who have under five-years-old children. Air quality improvement, fuel selection, and residential planning will help to reduce respiratory symptoms.

## Background

Poor air quality of the household is likely to be the largest human health concern in modern society [1]. In households with poor ventilation (as it is common in many low- and middle-income countries), exposures experienced by household members, particularly women and young children who spend a large proportion of their time indoors, have been documented to be many times higher to develop respiratory symptoms [2]. An increasing number of evidences have associated housing quality with morbidity and mortality from infectious diseases, chronic illnesses, injuries, poor nutrition, and mental disorders [3, 4]. Each year, close to 4 million people die prematurely from illness attributable to household air pollution from inefficient cooking practices using polluting stoves along with solid fuels such as dung, wood, agricultural residues, coal, and kerosene. Among these 4 million deaths, 27% are due to pneumonia, 18% from stroke, 27% from ischemic heart disease, 20% from chronic obstructive pulmonary disease (COPD), and 8% from lung cancer [5]. The magnitude of respiratory symptoms varies across countries. Evidence from a recent epidemiologic study conducted among adult residents in Guizhou province, China showed the prevalence of asthma-like symptoms, and asthma was 13.1% in winter [6]. The prevalence of respiratory symptoms among workers reported in Iran and Thailand varies from 15.5% [7] to 41% [8]. The self-reported respiratory symptom prevalence among women using traditional stoves in rural Honduras was 82% [9]. The prevalence of self-reported respiratory symptoms among Ethiopian women was 41.8% [10]. A study was done in Debre Berhan, Ethiopia demonstrated that the prevalence of bronchial asthma among adult patients was 29.6% [11]. Another comparative study carried out among textile factory workers in the northwest, Ethiopia revealed that the prevalence of self-reported respiratory symptoms in the slum areas of Addis Ababa and four rural kebeles of Butajira was 47.8% [12].

Poor housing conditions are associated with a wide range of health problems, including respiratory infections, asthma, lead poisoning, injuries, and mental health. It has a multitude of adverse health consequences on a woman as well. For instance, exposure to air pollutants during pregnancy can potentially impede fetal development and cause several ill birth outcomes such as intrauterine growth retardation, prematurity, abortion, low birth weight, congenital anomalies, and, in cases that are more severe, intrauterine or perinatal death [3, 13].

Among the diverse environmental concerns facing low- and middle-income countries including Ethiopia, housing is the most fundamental one. Most urban settings in low- and middle-income countries were not designed to handle millions of people which directly impact the availability and affordability of housing, forcing millions to live in substandard dwellings with poor housing quality [14]. Poor household air quality and housing, as well as crowding, are still typical basic problems of growing settlements and megacities, most of which are located in low- and middle-income countries [15].

However, in low- and middle-income countries, there are many relevant housing and health challenges still to be averted. Earlier studies have identified that different factors have been correlated with respiratory symptoms among mothers who have under five-year-old children. For instance: second-hand tobacco smoke, involvement in the charcoal production business, weaving, annual mean concentrations of NO_2_, total suspended particulates, particulates of less than 10 μm in aerodynamic diameter (PM10), ventilation, spinning, involvement in burning grass/field were significantly associated with respiratory symptoms [12, 16–18].

Evidences generated from this study would enable policymakers and program managers, administrators, Zonal health bureau, and other related stakeholders who work on this issue to understand the burden of the problem and its possible negative consequences which in turn direct them to a way of taking prompt actions and measures to alleviate the problem. The main objective of this study is to assess acute respiratory symptoms and its associated factors among mothers who have under five-year-old children in Gondar city, northwest Ethiopia.

## Methods and materials

### Study design, period, and area

**Fig. 1:**
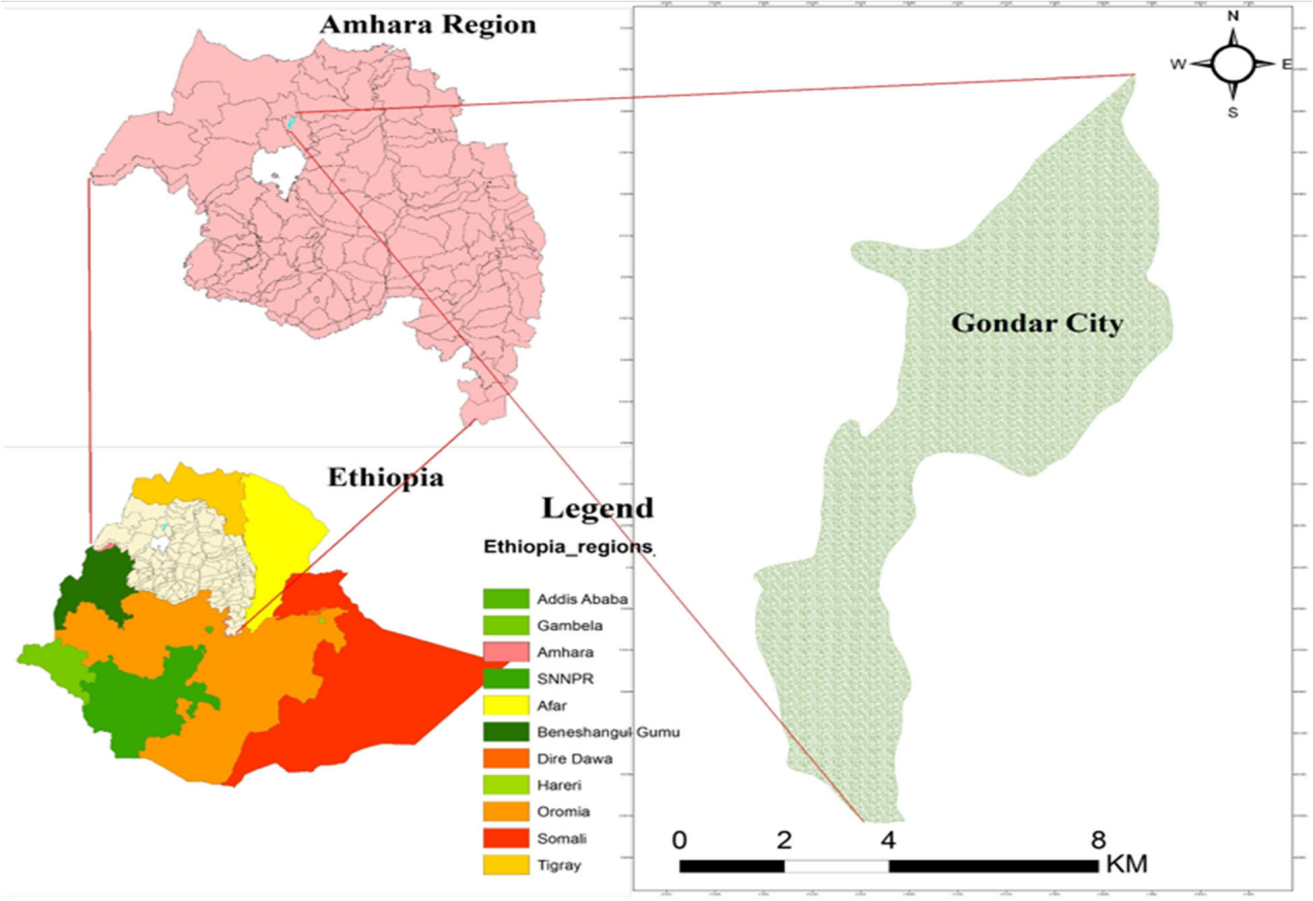
Map of study area

A community-based cross-sectional study was conducted from February 15, 2019 to June 20, 2019 in Gondar city, northwest Ethiopia. The city is located in Central Gondar Zone, Amhara Regional State of Ethiopia and is 748 km far from Addis Ababa, the capital city of Ethiopia. It is about 180 km from Bahir Dar city, the capital of Amhara Regional State. The city has an latitude of 12° 36′ N 37° 28′ E and longitude of 12.60° N 37.467° E with an elevation of 2133 m above sea level and is divided into 12 administrative areas (sub-cities), which consists of 22 kebeles (the smallest administrative units in Ethiopia) (Fig. 1). Gondar is among one of the ancient and largely populated cities in the country. It has an estimated total population of 324,000 with about 23,929 mothers of under-five years-old children.

### Sample size calculation and sampling procedure

The sample size was determined by using a single population proportion formula considering the following assumptions: *p* = 50% proportion of mothers with respiratory symptoms (there was no previous study in the study area at the study period), 95% confidence interval, 5% margin of error (*d*) and design effect 2

$$ n=\frac{{\left({Z}_{\frac{\alpha }{2}}\right)}^2\times p\left(1-p\right)}{d^2}\kern2.25em n=\frac{(1.96)^2\times 0.5\left(1-0.5\right)}{(0.05)^2}=384 $$

By taking 5% of the non-response rate, then the total sample size was 806.

**Fig. 2:**
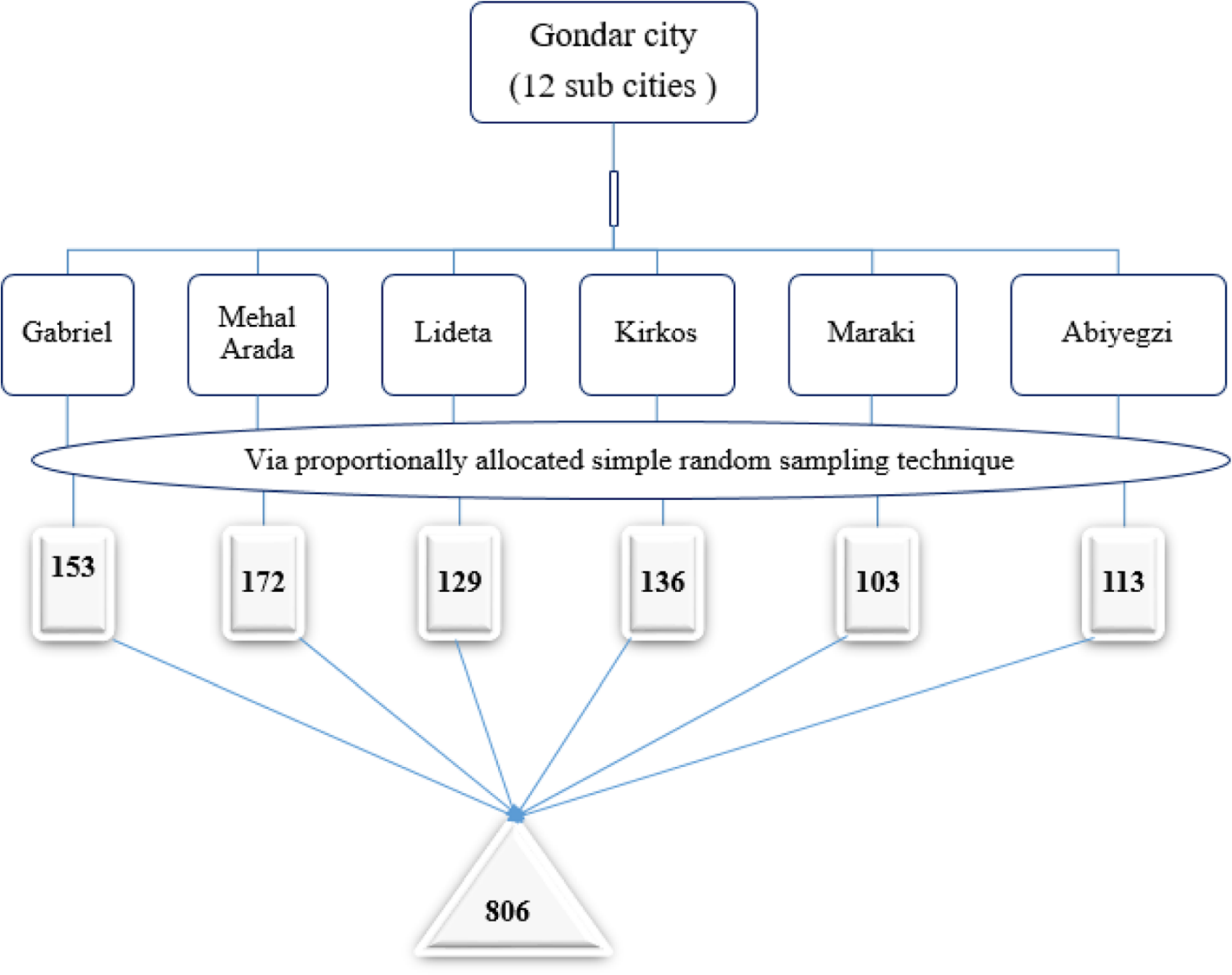
A flow chart of the sampling procedures for selection of study participants in Gondar city, northwest Ethiopia 2019

Multi-stage sampling technique was used as an assumption of being a heterogeneous population in the 12 administrative areas of the cities. Fifty percent of total sub-cities were selected through lottery method from the 12 administrative areas and all eligible study participants in the selected sub-cities were included in the study (Fig. 2).

### Data collection tools and procedures

Through a face-to-face interview at the participants’ home, data were collected using a pretested semi-structured questionnaire (Additional file 1). The questionnaire was pre-tested to check the response, language clarity, and appropriateness at Azezo Dimaza sub-city (outside of the study area) with 5% of the total sample size (on 41 women). Based on the findings from the pre-test, modification and arrangement of questions was done. The outcome variable, i.e. respiratory symptoms (such as cough, shortness of breathing, wheezing, chest tightness, phlegm, and blocked or running nose); the explanatory variables such as sociodemographic factors (age of mothers, average monthly income, mother and spouse educational level, etc.) and household air pollutants (such as type fuels for cooking, types of fuel for heating, pesticide application, contact with farm animals (e.g., cattle, pigs, goats, sheep or poultry), smoke cigarettes (mothers/spouse), cockroach infestation in the household, painting/staining done in the last 6 months in households (HH), new carpet, drapes or other textiles in the last 6 months, and using of air fresheners, etc.); and house characteristics such as floor construction materials, wall surface water-based paint, ceiling surface, damp stains, visible mold, normal cooking done, open doors during cooking, open windows during cooking, and time spent indoors on an average day were included in the questionnaires.

The questionnaire was first prepared in English and then translated to Amharic (local language) and back to English to maintain consistency of the tool. Six diploma nurses for data collection and one BSc nurse for supervision were involved after a two-day training.

### Operational definitions

The outcome variable of this study was respiratory symptoms. Respiratory symptoms were defined as whether mothers had been suffering from cough, shortness of breathing, wheezing, chest tightness, phlegm, and blocked or running nose in the past 12 months [19, 20]. Mothers who have experienced at least one of the abovementioned symptoms were considered as having respiratory symptoms.

### Data processing and analysis

Data were first checked manually for completeness and then coded and entered into Epi Info version 7.1.2.0. Then the data were exported to Stata 14.00 for data checking, cleaning, and analysis. Descriptive statistics were performed to describe the study population in relation to dependent and independent variables. Model fitness was checked with the assumptions of the Hosmer and Lemeshow test. Bivariable and multivariable logistic regressions were computed to identify the presence and strength of association. Variables with a *p* value < 0.2 during the bivariable binary logistic regression analysis were included in the multivariable binary logistic regression analysis. Odds ratio with 95% CI was computed and variables having a *p* value less than 0.05 in the multivariable binary logistic regression model were considered significantly associated with the dependent variable. Variance inflation factor was done to test multicollinearity (Additional file 2). To report this study Strengthening the Reporting of Observational Studies in Epidemiology (STROBE) guideline was used (Additional file 3).

## Result

### Sociodemographic characteristic of the study participants

A total of 806 study participants were aimed in this study. Of this, 792 participants were enrolled with a response rate of 98.26%. Two hundred twenty-seven (28.65%) mothers were in the age category of 18–25 years whereas one hundred eighty-two (22.98%) were in the class of above 33 years. The majority (77.02%) of study participants were Orthodox Christian in religion followed by Muslims (17.68%). Two thirds of mothers (67.98%) were housewives (Table 1).

**Table 1:** Sociodemographic characteristics of study participants in Gondar city, northwest Ethiopia, 2019

Variables	Frequency (*n* = 792)	Percent (%)
Education level of mothers (*n* = 778)		
Unable to read and write	121	15.55
Read and write	90	11.57
Primary	100	12.85
Secondary	253	32.52
Graduate from vocational	30	3.86
Diploma and above	184	23.65
Age of mothers		
18–25 years	227	28.66
26–28 years	206	26.01
29–32 years	177	22.35
≥ 33 years	182	22.98
Median age of mothers 28 ± 5.86 (SD)		
Religion		
Orthodox	610	77.02
Muslim	140	17.68
Others*	42	5.30
Mother’s occupation (*n* = 784)		
Housewife	533	67.98
Farmer	4	0.51
Student	8	1.02
Private employee	52	6.63
Government employee	142	18.11
Merchant	35	4.46
Others (specify)	10	1.28
Occupation of spouse (*n* = 723)		
Farmer	18	2.49
Student	8	1.11
Private employee	310	42.88
Government employee	232	32.09
Merchant	88	12.17
Others	67	9.27

* Protestants, Jewish

### Household air pollution and housing characteristics

Three- fifth (60.48%) and one- third (34.47%) of the study participants used charcoal and electricity for cooking food, respectively. Nearly half (47.85%) of the households were infested by cockroaches. Of the study participants, 250 (31.57%) spend 6 to 11 h in their house/home, while 198 (25%) mothers spend their time in the household for about < 6 h. Above half (55.56%) of the study participants used wood and coal for heating their house during humid conditions and 43.06% did not use any fuel for heating (Table 2).

**Table 2:** Household air pollution and housing characteristics of the study participants in Gondar city, northwest Ethiopia, 2019

Variables		Frequency (*n* = 792)	Percent (%)
Types of fuel usually used for cooking			
Charcoal		479	60.48
Electricity		273	34.47
Open fires		40	5.05
Types of fuel usually used for heating			
None		341	43.06
Wood, coal		440	55.56
Electricity		11	1.39
Contact with farm animals (e.g., cattle, pigs, goats, sheep, or poultry)			
No		745	94.07
Yes		47	5.93
Smoke cigarettes (mothers/guardians)			
No		768	96.97
Yes		24	3.03
Cockroach infestation in household			
No		413	52.15
Yes		379	47.85
Painting/staining done in the last 6 months HH			
No		682	86.11
Yes		110	13.89
New carpet, drapes, or other textiles in the last 6 months			
No		682	86.11
Yes		110	13.89
Using air freshener			
No		744	93.94
Yes		48	6.06
Presence of a kitchen exhaust fan			
No		752	94.95
Yes		40	5.05
A pesticide application			
No		635	80.18
Yes		157	19.82
Floor construction materials			
Wood and mud		542	68.43
Brick and concrete		250	31.57
Wall surface water-based paint			
No		618	78.03
Yes		174	21.97
Ceiling surface (*n* = 749)			
Wooden		576	76.90
Painted		131	17.49
Cement		42	5.61
Damp stains			
No		729	92.05
Yes		63	7.95
Visible mold			
No		725	91.54
Yes		67	8.46
Place of cooking (*n* = 775)			
Inside		461	59.48
Outside		314	40.52
Open doors during cooking			
No		439	64.56
Yes		241	35.44
Open windows during cooking			
No		462	68.34
Yes		214	31.66
Average length/duration of time spend in house/home/indoor on a day			
< 6 h		198	25.00
6 to 11 h		250	31.57
11 to 15 h		165	20.83
> 15 h		179	22.60
Presence of garage less than 100 m from household			
No		746	94.19
Yes		46	5.81
Frequency of trucks passing through the street where you live, on weekdays			
Never		397	50.13
Seldom		254	32.07
Frequently		93	11.74
Almost the whole day		48	6.06
Living less than 100 m heavy traffic			
No		630	79.55
Yes		162	20.45
Living less than 100 m (unpaved roads/streets)			
No		598	75.51
Yes		194	24.49
Exposure to animal allergens in early childhood			
No		754	95.20
Yes		38	4.80
Exposure to animal allergens in the present time			
No		747	94.32
Yes		45	5.68

### Prevalence of respiratory symptoms among mothers

**Fig. 3:**
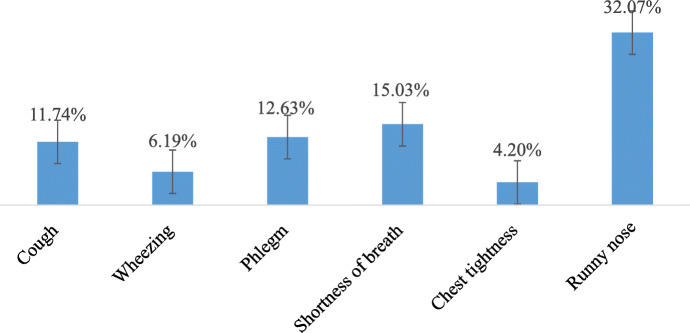
Prevalence of respiratory symptoms among mothers in Gondar city, northwest Ethiopia, 2019

The prevalence of respiratory symptoms among mothers in Gondar city was 46.1% at 95% [CI: 42.6%–49.7%]. Commonly reported respiratory symptoms were runny nose, shortness of breath, and phlegm with a magnitude of 32.07%, 15.03%, and 12.63%, respectively. The lowest recorded respiratory symptom among mothers was chest tightness which accounted for 4.42% (Fig. 3).

### Factors associated with respiratory symptoms of mothers

On multivariable analysis, educational level of mothers; poor (dusty or smelly) working environment; frequent use of fuel for cooking and heating; painting/staining done in the last 6 months HH; new carpet, drapes, or other textiles in HH; pesticide use at HH; floor construction materials; damp stains; length/duration of time spending in the house/ indoors; and living less than 100 m (unpaved roads/streets) were significantly associated with mothers’ respiratory symptoms.

Study participants who graduated from vocational training were 74% less likely to encounter respiratory symptoms compared to those who cannot or are unable to read and write (AOR = 0.26 at 95% CI: 0.08–0.82). Mothers who work in a poor (dusty or smelly) environment were 2.90 times more likely to develop respiratory symptoms than their counterparts (AOR = 2.90 at 95% CI: 1.39 – 6.08).

Study participants who used an open fire for cooking were 63% less likely to develop respiratory symptoms compared with those who used charcoal (AOR = 0.37 at 95% CI: 0.16–0.85). Mothers who live in painted HH were 1.95 times more likely to develop respiratory symptoms than their counterparts (AOR = 1.95 at 95% CI: 1.03–3.69).

Those respondents who use new carpets in HH were 2.02 times more likely to develop respiratory symptoms compared with their counterparts (AOR = 2.02 at 95% CI: 1.08–3.77). Mothers who used pesticides in HH were 1.71 times at higher risk of experiencing respiratory symptoms than those who did not use (AOR = 1.71 at 95% CI: 1.03–2.84).

Those respondents, whose house floor was constructed from brick and concrete were 47% times less likely to be a victim of respiratory symptoms when compared with those who constructed their house floor from mud and wood (AOR = 0.53 at CI: 0.32–0.89). The odds ratio of having respiratory symptoms among mothers whose households had damp stains was 2.45 compared to their counterparts (AOR = 2.45 at 95% CI: 1.04–5.75).

Study participants who spend much of their time (6 to 11 h) indoors were 2.59 times highly likely to face respiratory symptoms compared with those who spent a few hours (< 6 h) indoors (AOR = 2.59 at 95% CI: 1.53–4.37) and the risk increases among respondents who spent 11 to 15 h of their time in the house/indoors (AOR = 3.47 at 95% CI: 1.87–6.43).

Study participants who live less than 100 m (unpaved roads/streets) were 4.35 times more likely to experience respiratory symptoms than their counterparts (AOR = 4.35 at 95% CI: 2.64–7.18) (Table 3).

**Table 3:** Factors associated with mothers’ respiratory symptoms in Gondar city northwest Ethiopia, 2019

Variables	Respiratory symptom		COR 95% CI	AOR 95% CI
	Absent	Present		
Educational level of mothers				
Unable to read and write	56	65	1	1
Read and write	32	58	1.56(0.89–2.73)	1.24(0.63–2.49)
Primary	58	42	0.62(0.37–1.06)	0.69(0.35–1.37)
Secondary	141	112	068(0.44–1.06)	0.57(0.32–1.02)
Graduate from vocational training	22	8	0.31(0.13–0.75)	0.26(0.08–0.82)*
Diploma and above	110	74	0.51(0.36–0.92)	0.55(0.29–1.05)
Poor (dusty or smelly) working environment				
No	403	319	1	1
Yes	24	46	2.44(1.45–4.05)	2.90(1.39–6.08)*
Fuel type usually used for cooking				
Charcoal	237	242	1	1
Electricity	164	109	0.65(0.48–0.88)	0.73(0.45–1.16)
Open fires	26	14	0.53(0.26–1.03)	0.37(0.16–0.85)*
Fuel type usually used for heating				
None	222	119	1	1
Wood, coal	201	239	2.22(1.66–2.97)	1.17(0.76–1.81)
Electricity	4	7	3.26(0.94–11.37)	1.77(0.42–7.40)
Mother contact with farm animals (e.g., cattle, sheep, or poultry)				
No	413	332	1	1
Yes	14	33	2.93(1.54–5.56)	1.41(0.65–3.07)
Painting/staining been done in the last 6 months HH				
No	390	292	1	1
Yes	37	73	2.63(1.73–4.02)	1.95(1.03–3.69)*
New carpet, drapes, or other textiles in HH				
No	39	288	1	1
Yes	33	77	3.19(2.07–4.93)	2.02(1.08–3.77)*
Pesticide use at HH				
No	366	269	1	1
Yes	61	96	2.14(1.49–3.06)	1.71(1.03–2.84)*
Floor construction materials				
Wood and mud	263	279	1	1
Brick and concrete	164	86	0.49(0.36–0.67)	0.53(0.32–0.89)*
Wall surface water-based paint				
No	312	306	1	1
Yes	115	59	0.52(0.37–0.74)	0.81(0.46–1.41)
Damp stains				
No	402	327	1	1
Yes	25	38	1.86 (1.10–3.16)	2.45(1.04–5.75)*
Open windows during cooking				
No	207	255	1	1
Yes	138	76	0.44(0.32–0.62)	0.71(0.45–1.13)
Duration/length of time spending indoors				
< 6 h	127	71	1	1
6 to 11 h	121	129	1.90(1.30–2.79)	2.59(1.53–4.37)**
11 to 15 h	65	100	2.75(1.79–4.21)	3.47(1.87–6.43)**
> 15 h	114	65	1.02(0.67–1.55)	1.65(0.83–3.29)
Presence of attached garage living less than 100 m HH				
No	414	332	1	1
Yes	13	33	3.16(1.64–6.11)	1.91(0.85–4.25)
Trucks pass through the street where you live, on weekdays?				
Never	255	142	1	1
Seldom	103	151	2.63(1.90–3.63)	1.52 (0.94–2.44)
Frequently	43	50	2.08(1.32–.29)	1.01(1.87–6.43)
Almost whole day	26	22	1.51(0.83–2.77)	0.66(0.83–3.29)
Living less than 100 m from heavy traffic				
No	366	264	1	1
Yes	61	101	2.29 (1.60–3.27)	1.25(0.73–2.15)
Living less than 100 m (unpaved roads/streets)				
No	382	216	1	1
Yes	45	149	5.85(4.03–8.50)	4.35(2.64–7.18)**
Exposure to animal allergens in early childhood				
No	415	339	1	1
Yes	12	26	2.65(1.32–5.33)	0.51(0.13–1.98)
Exposure to animal allergens in the present time				
No	411	336	1	1
Yes	16	29	2.21(1.18–4.15)	1.19(0.36–3.85)

1 = Reference group

* Significant at *p* < 0.05, ** Significant at *p* < 0.001

## Discussion

The overall prevalence of respiratory symptoms in the current study was 46.1% at 95% [CI: 42.6%–49.7%]. This result is lower than self-reported respiratory symptoms from rural Honduras [9] and South India [21]. However, the prevalence was higher than respiratory involvement among women exposed to the smoke of traditional biomass and gas fuel in Bangladesh [22], respiratory symptoms in Indian women [23], and women in West Sierra Leone [24]. The prevalence is closer to an earlier report from Ethiopia [10]. The differences in the proportion of self-reported respiratory symptoms may be attributed to a difference in the level of socioeconomic status, housing conditions, fuel type used, measurement difference, and study period.

Educational status was associated with mothers' respiratory symptoms. Mothers who attended technical and vocational training were less likely to develop respiratory symptoms as compared to those who cannot read and write. Lower educational status was reported as a risk factor for asthma and respiratory symptoms in many cross-sectional studies [25–28] and a cohort study [29]. The reason for the association of educational status and respiratory symptoms might be due to the fact that educational status is among the most common indicators used for measurement of socioeconomic status [30]. Educational level was associated with income and the capacity to buy clean fuel in a previous study [31] and lower educational level was more strongly associated with biofuel use which results in a higher risk of respiratory infection [32, 33].

In our analysis, the adjusted odds ratio of respiratory symptom was 2.9-folds among mothers working in dusty and smelly environment compared to those who reported working in a clean environment. The effects of occupational dust exposure on the reduction of pulmonary function and aggravation of respiratory symptoms have long been confirmed [34–36]. Exposure to dust results in a reduction of lung function due to pulmonary obstruction, and dust particles serve as a vehicle for disease-causing microorganisms [35, 36]. The chemical composition of dust may also be responsible for respiratory symptoms [37, 38].

Fuel type usually used for cooking is another factor associated with respiratory symptoms in this study. The use of open firewood was protective against respiratory symptoms as compared to charcoal use in the current study. This is against reports from several earlier evidence [39, 40], whereby charcoal was better than an open fire and firewood in terms of a respiratory outcome as coal produces less particulate matter than open fire and wood. Even though charcoal is often considered as a clean fuel compared to the other biomass fuels, such as firewood, animal dung, crop residues [41, 42], Das et al. [39] and Sana et al. [43], consistent to the present study, found positive associations between respiratory symptoms and firewood compared to charcoal. Sana et al. [43] reported that charcoal is not easy to set on fire, and the cooks usually use fire starters such as plastic bags, tire rubber cut or inner tire tubes, drainage oil, paper, agricultural waste, petrol coke powder, twigs, and dry herbs. Plastic especially releases toxic gases like dioxins, furans, mercury, and polychlorinated biphenyls, as well as some additives as phthalates and brominated flame retardants which pose respiratory health risks [44].

Mothers who reside at houses painted in the past 6 months prior to the study were at higher odds of respiratory symptoms. Indoor paints may emit harmful chemicals that result in respiratory irritation [45]. Recent indoor painting was reported as one of the risk factors to respiratory symptoms in several previous studies [45–50].

Study participants who live in houses and reported pesticide use were at higher risk of respiratory symptoms. This was in line with an earlier study [51]. The potential plausibility of the observed association can be explained by the toxicological and clinical nature of the pesticides. Most pesticides have low molecular weight, which enables them to induce immediate humoral immune-mediated allergic reactions [52, 53]. Exposure to organo chlorines at high concentrations may result in mucus hypersecretion and airway smooth muscle contraction leading to breathlessness, wheezing, and cough [54]. It favors the T-helper cell (Th) type 2 immunophenotype associated with asthma and allergy [55].

Living within 100 m of the unpaved road was a risk factor for respiratory symptoms in the sense of exposure to outdoor air pollutants. There is well-established evidence that exposure to air pollution results in a higher risk of a respiratory infection such as pneumonia [56–61].

The presence of damp stain was another risk factor affecting respiratory symptoms, and this is not surprising as mold growth is favored in damp conditions [62, 63]. In the current study, the presence of new carpet in the house was a risk factor for respiratory symptoms among women. The presence of new furniture such as carpet has been associated with respiratory illnesses in several earlier studies [64, 65]. Carpets harbor house dust and thereby respiratory illness-causing pathogens [66].

Respondents who spend much of their time (6 to 11) hours and (11 to 15) hours indoors had 2.59 and 3.47 high odds of facing respiratory symptoms compared with those who spent a few hours (< 6 h), respectively. Even though the current study has shown spending a long time in indoor environments is a risk for acute respiratory symptoms, this is not consistent with spending more than 15 h indoors. Perhaps, those who spend their time indoors may have kept the house and ventilation and their surrounding environments clean.

Lastly, study participants who live in houses made up of wood and mud were at higher risk of respiratory symptoms.

## Limitations of the study

Although large sample size and simple random sampling employed in this study which helps for greater generalizability, lack of measurements, i.e., pulmonary function test and inherent nature of the cross-sectional study which fails to show cause–effect relationships were the limitations of this study. In addition, recall and social desirability biases might be high in such types of self-reported cross-sectional studies.

## Conclusion

The overall prevalence of respiratory symptoms was relatively high among mothers who have under five-years-old children. Several modifiable factors such as educational level, working in a dusty environment, fuel type used for cooking, recent house painting, presence of new carpet and damp stain, pesticide use, living in mud- and wood-walled houses, spending longer time indoors, and living near unpaved roads/streets were found to be significantly associated with respiratory symptoms among mothers. Thus, we recommend policymakers and implementers to engage in household air quality improvements, fuel selection, and residential house planning to help mothers protect from respiratory symptoms.

## Supplementary information

**Additional file 1.** Questionaire.

**Additional file 2.** VIF result.

**Additional file 3.** STROBE checklist.

## Data Availability

The dataset analyzed during the current study are available from the corresponding author on reasonable request.

## Abbreviations

AOR: Adjusted odds ratio
CI: Confidence interval
COR: Crude odds ratio
HH: Household
