# Supplementary material for: Acute respiratory symptoms and its associated factors among mothers who have under five-years-old children in northwest, Ethiopia
Source: Environ Health Prev Med. 2020 Jun 15;25:20. doi: 10.1186/s12199-020-00859-4 (PMC7296770; doi:10.1186/s12199-020-00859-4)
Supplement: Supplementary file 1 — Additional file 1. Questionaire. [file 12199_2020_859_MOESM1_ESM.pdf]

Information Sheet

Title: "Acute respiratory symptoms and its associated factors among mothers who have under five-year-old children in northwest, Ethiopia"

Name of principal investigator: - Zewudu Andualem

Name of the organization: - University of Gondar, College of Medicine and Health Sciences, Institute of Public Health.

Introduction

My name is ____________ and I am a data collector. Right now I am going to give you the relevant information concerning the research and I invite you to be part of this research. Before you decide to be part of the research you can talk anyone to feel comfortable with the research. If there is any word that you do not understand while I am giving the information, please stop me and ask me and I will explain to you. The aim of the project to assess acute respiratory symptoms and its associated factors among mothers who have under five-year-old children in northwest, Ethiopia. There is no risk or direct benefit in participating in this research project. You will not be provided any incentive or payment to take part in this project. Participating and not participation is the full right of participant and they can stop participating in the study at any time. They can also skip any question which they don't want to respond. They can ask any question which is not clear for them. It will take 30 minutes.

Are you voluntary to participate?

☐ Yes (say thank you, continue with her) ☐ No (Say thank you leave her)

Person to contact: - If you have any question you can contact principal investigator at any time

Zewudu Andualem (PI, University of Gondar), Phone +251918570249:

Email: zewuduandualem12@gmail.com

Questionnaire code _________________________________Starting time__________________

Name of sub city _________________________________House hold number _______________

Name of data collector_____________________________________Sign._________________

Socio - demographic factors mothers who have under five-year-old children				
1.	Number of people living in the same household ______________________			
2.	Age of mothers ________________years			
3.	Religion	1) Orthodox<br>2) Muslim	3) Protestant<br>4) Other's specify_____________	
4.	Marital staus of mothers/gurdians	1. Single<br>2. Married	3. Divorced<br>4. Widowed	5. Separated

5.	Education level of the mother/	1. Unable to read and write<br>2. Read and write<br>3. Primary	4. Secondary<br>5. Graduate from vocational<br>6. Diploma and above	
6.	Education level of the spouse	1) Unable to read and write<br>2) Able to read and write<br>3) Primary	4) Secondary<br>5) Graduate from vocational<br>6) Diploma and above	
7.	Average monthly income	____________________________ETB		
8.	Occupation of mother	1) House wife<br>2) Farmer<br>3) Student	4) Private employee<br>5) Government employee	6) Merchant<br>7) Others (specify) __
9.	Occupation of spouse	1) Farmer<br>2) Student	3) Private employee<br>4) Government employee	5) Merchant<br>6) Others (specify)___________
	**Mothers respiratory symptoms**			
	**Cough**			
1.	Do you usually have a cough?	0. No	1. Yes	**If "No", go to Q4**
2.	Do you usually cough like this on most days for the last months or more during the year?	0. No	1. Yes	
3.	For how many days/months/years have you had this cough?	Number of days/months/years_______		
	**Wheezing**			
4.	Do you usually have wheezing	0. No	1. Yes	**If "No" go to Q7**
5.	Mostly do you have wheezing	0. In days	1. In nights	
6.	Have you ever required medicine or treatment? For the attack(s)?	0. No	1. Yes	
	**Breathlessness**			
7.	Have your child ever had phlegm	0. No	1. Yes	
8.	Have your child ever had pneumonia? Symptoms include fever, cough, tiredness (fatigue), and chest pain.	0. No	1. Yes	
9.	Have you ever had Asthma	0. No	1. Yes	
10	Have you ever had Acute Respiratory Infection diagnosis	0. No	1. Yes	
11				
12	Has your child ever had tonsillectomy	0. No	1. Yes	
13	Has your child ever had **hay fever**	0. No	1. Yes	
14	Has your child ever had eczema?	0. No	1. Yes	
15	Has had or ever had tuberculosis?	0. No	1. Yes	
16	Have you ever had troubled by shortness of breath? **0. No** **1. Yes**<br>**Grade 0: No breathlessness except with strenuous exercise**			

	**Grade 1: Breathlessness when hurrying on the level ground or walking up a slight hill at least one months in a year**<br>**Grade 2: Walking slower than people of the same age on the same level because of breathlessness or need to stop for breath when walking at their own pace or level at least one months in a year**<br>**Grade 3: Stopping for breath after walking about a certain distance or a few minutes on the level ground at least one months in a year**<br>**Grade 4: Being too breathless to leave the house or breathless when dressing or undressing at least one months in a year**				
17	Have your child ever had chest tightness		0. No		1. Yes
18	Have your child ever had bronchitis		0. No		1. Yes
19	Have your child ever had Emphysema		0. No		1. Yes
	Occupational history				
20	Have you worked in the last 5 years in any poor indoor or outdoor (dusty or smelly) environment?		0. No	1. Yes<br>If yes , please specify ________	
	Tobacco smoking				
21	Does any member of this household smoke?		0. No	1. Yes	**If "No" go to 23**
22	What types of tobacco do you smoke?				
	Cigarettes		0. No	1. Yes	
	Cigars (local)		0. No	1. Yes	
23	Do they usually smoke indoors?		0. No	1. Yes	
	Mothers family health history				
24	Mother	Does have lung cancer?	0 No	1 Yes	2 Don't know
		Chronic bronchitis?	0. No	1 Yes	2 Don't know
		Emphysema?	0. No	1 Yes	2 Don't know
		Asthma?	0. No	1 Yes	2 Don't know
25	Father	Does have lung cancer?	0. No	1 Yes	2 Don't know
		Chronic bronchitis?	0. No	1 Yes	2 Don't know
		Emphysema?	0. No	1. Yes	2. Don't know
		Asthma?	0. No	1 Yes	2. Don't know
	**Potential associated factors with respiratory symptom**				
	**I. Indoor air pollution**				
1.	In your house, what fuel is usually used for cooking?		1. Charcoal<br>2. Electricity<br>3. Gas	4. Open fires<br>5. Other______________	
2.	In your house, what fuel is usually used for heating		1. None	4. Gas, kerosene, paraffin	

		2. Wood, coal, oil<br>3. Electricity	5. Other – Please specify ________________
3.	Has this child's Mother had regular (at least once a week) contact with farm animals (e.g. cattle, pigs, goats, sheep or poultry) while being pregnant with this child? **0. No** **1. Yes**		
4.	Does child mother (or female guardian) smoke cigarettes?<br>0. No 1. Yes		If YES, how many cigarettes smoke each day? ______________________
5.	Does child's father (or male guardian) smoke cigarettes?<br>0. No 1. Yes		If YES, how many cigarettes smoke each day? ___________________
6.	How many people living in the house smoke cigarettes _____________people including parents?		
7.	Is there Cockroach infestation in household	0. No	1. Yes
8.	Has the building ever had a fire?	0. No	1. Yes When_______________
9.	Is there smoking in the building?	0. No	1. Yes How frequently? ________
10	Has painting/staining been done in the last 6 months HH?	0. No	1. Yes
11	Is there new carpet, drapes or other textiles in the last 6 months?	0. No	1. Yes
12	Have air fresheners been used recently in the last one weeks?	0. No	1. Yes
13	Is there a kitchen exhaust fan	0. No	1. Yes
14	Is there a clothes dryer?	0. No	1. Yes
15	Has there been a pesticide application?	0. No	1. Yes

<u>Checklist</u>: Household characteristics

S.No	Variable			
1)	Age of buildings _______________	7) Cleaning frequency house _________per day/week		
2)	No of beds per HH_________________	8) No functional windows HH _________________		
3)	Floor area (m^2)______________________	Density of occupant (m^3/occupant)_____________		
4)	How many rooms are there in your home __________________________			
	Questions 5 to 14 interviewer should be observe and measure			
9)	Floor construction materials (multiple answer possible)	0. Wooden<br>1. Mud	2. Concrete<br>3. Brick	4. Others (Specify) ________________
10)	Wall surface Water based paint	0. No	1. Yes	
11)	Ceiling surface	0. Wooden	1. Painted	
12)	Damp stains	0. No	1. Yes	
13)	Visible mould	0. No	1. Yes	
14)	Where is normal cooking done?	1. Inside	2. Outside	
15)	What type of material is the kitchen floor made from?	1. Clay & mud	2. Cement	3. Other (specify)_____

16)	What type of roof (material of construction?) does the kitchen level?	1. Thatch<br>2. Iron sheets	3. Asbestos	4. Other specify ______________
17)	What type of wall (material of construction) is the kitchen made of?	1. Thatch<br>2. Wood	3. Mud & wood<br>4. Stone or brick	5. Other specify ___________
18)	Number of windows in the room in which cooking takes place_________________			
19)	Do you normally open doors during cooking?		0. No	1. Yes
20)	Do you normally open windows during cooking? (assess good or bad ventilation)		0. No	1. Yes
21)	Is there any poor outdoor air influence during cooking?		0. No	1. Yes
22)	How many people are normally present in the kitchen when cooking is being done? ___________			
23)	Who is normally present during cooking times in the kitchen?	1. Mother<br>2. Mother & children less than 5yrs	3 Mother & children<br>4 Whole families<br>5 Other______________	
24)	What type of stove is used for cooking (in terms of combustion chamber)?	1 Open combustion<br>2 Partly open combustion<br>3 Enclosed chamber with no flue	4 Enclosed chamber with a flue<br>5 Other Specify _________	
25)	How much time on average does cooking take per day? _____________ (hrs.)			
26)	How much time do you spend indoors on an average day? ___________ (hrs.)			
	**Outdoor air pollution**			
27)	Is there an attached garage Living less than 100 m house hold?		**0.** No	**1.** Yes
28)	How often do trucks pass through the street where you live, on weekdays?	1. Never<br>2. Seldom	3. Frequently through the day<br>4. Almost the whole day	
29)	Living less than 100 m Heavy traffic	0. No	1. Yes	
30)	Living less than 100 m chimneys	1. No	2. Yes	
31)	Living less than 100 m recycling warehouses	0. No	1. Yes	
32)	Living less than 100 m (Unpaved roads/streets)	0. No	1. Yes	
33)	Exposure to animal allergens in early childhood (regarding child)		0. No	1. Yes
34)	Exposure to animal allergens in present time		0. No	1. Yes

"Thank you for your participation"
