# Supplementary material for: Acute respiratory symptoms and its associated factors among mothers who have under five-years-old children in northwest, Ethiopia
Source: Environ Health Prev Med. 2020 Jun 15;25:20. doi: 10.1186/s12199-020-00859-4 (PMC7296770; doi:10.1186/s12199-020-00859-4)
Supplement: Supplementary file 3 — Additional file 3. STROBE checklist. [file 12199_2020_859_MOESM3_ESM.pdf]

Variable	VIF	1/VIF
N7Educatio~i	**1.23**	**0.811333**
1.N19Havey~i	**1.08**	**0.926103**
N1Inyourho~s		
2	**1.38**	**0.723314**
3	**1.08**	**0.926044**
4	**1.15**	**0.872550**
N2Inyourho~s		
2	**1.19**	**0.841494**
3	**1.08**	**0.926458**
1.N3Hasthi~a	**1.12**	**0.893808**
1.N10Haspa~n	**1.52**	**0.657504**
1.N11Isthe~r	**1.47**	**0.678806**
1.N15Hasth~a	**1.11**	**0.903410**
1.N11Walls~t	**1.38**	**0.725771**
1.N13_Damp~s	**1.17**	**0.851742**
1.N21Doyou~n	**1.31**	**0.764906**
1.N28Isthe~g	**1.10**	**0.906051**
N29Howofte~h		
1	**1.43**	**0.700877**
2	**1.37**	**0.728007**
3	**1.46**	**0.682947**
1.N30Livin~i	**1.48**	**0.673459**
1.N33Livin~d	**1.24**	**0.807360**
1.N34Expos~a	**1.82**	**0.548294**
1.N35Expos~r	**1.86**	**0.536682**
Mean VIF	**1.32**	
